# *Carpesium divaricatum* Sieb. & Zucc. Revisited: Newly Identified Constituents from Aerial Parts of the Plant and Their Possible Contribution to the Biological Activity of the Plant

**DOI:** 10.3390/molecules24081614

**Published:** 2019-04-24

**Authors:** Natalia Kłeczek, Barbara Michalak, Janusz Malarz, Anna Karolina Kiss, Anna Stojakowska

**Affiliations:** 1 Institute of Pharmacology, Polish Academy of Sciences, Department of Phytochemistry, 31-343 Kraków, Smętna Street 12, Poland; kleczek@if-pan.krakow.pl (N.K.); malarzj@if-pan.krakow.pl (J.M.); 2 Department of Pharmacognosy and Molecular Basis of Phytotherapy, Medical University of Warsaw, 1 Banacha Street, 02-097 Warsaw, Poland; bmichalak@wum.edu.pl (B.M.); akiss@wum.edu.pl (A.K.K.)

**Keywords:** anti-inflammatory, *Carpesium divaricatum*, caffeic acid derivatives, cytokines, 12-oxo-phytodienoic acid, ROS

## Abstract

*Carpesium divaricatum* Sieb. & Zucc. has a long history of use as both a medicinal and a food plant. However, except for terpenoids, its chemical constituents have remained poorly investigated. The composition of hydroalcoholic extract from aerial parts of *C. divaricatum* was analyzed by HPLC-DAD-MS^n^, revealing the presence of numerous caffeic acid derivatives that were formerly unknown constituents of the plant. In all, 17 compounds, including commonly found chlorogenic acids and rarely occurring butyryl and methylbutyryl tricaffeoylhexaric acids, were tentatively identified. Fractionation of lipophilic extract from cultivated shoots led to the isolation of 12-oxo-phytodienoic acid (12-OPDA), which is a newly identified constituent of the plant. The compound, at concentrations of 0.5, 1.0, and 2.5 μM, significantly reduced IL-8, IL-1β, TNFα, and CCL2 excretion by lipopolysaccharide (LPS)-stimulated human neutrophils. Reactive oxygen species (ROS) production induced by f-MLP was also significantly diminished in the neutrophils pretreated by 12-OPDA. The newly identified constituents of the plant seem to be partly responsible for its pharmacological activity and elevate the value of *C. divaricatum* as a potential functional food.

## 1. Introduction

The genus *Carpesium* (Asteraceae, tribe Inuleae), following recent taxonomic studies, comprises 25 species distributed in Asia and southeastern Europe [1]. Plants of the genus, due to their anti-inflammatory, antipyretic, detoxifying, anti-oedematous haemostatic, and antimalarial properties, have long been used as traditional medicines in China, Japan, and Korea to treat various ailments [2,3]. Whole herbs or aerial parts of the plants are also eaten, by local communities, as a seasonal vegetable, functional food, or vitamin source [4,5]. Aerial parts of *Carpesium* plants are also used to prepare brewed beverages or serve as forage [2,5].

The whole plant of *Carpesium divaricatum* Sieb. & Zucc. has been used in traditional medicine of the Far East as a remedy in colds, fevers, diarrhea, infections of the urinary tract, sore throat, toothache, and ascaridiasis [3]. Phytochemical studies on specialized metabolites of the plant are almost entirely devoted to terpenoids, especially sesquiterpene lactones, which reportedly possess anti-inflammatory and cytostatic properties [3,6,7].

The aim of the present study was an identification of non-terpenoid constituents of the plant that may contribute to the activity of its preparations.

## 2. Results

### 2.1. Caffeic Acid Derivatives in Aerial Parts of C. divaricatum

**Figure 1:**
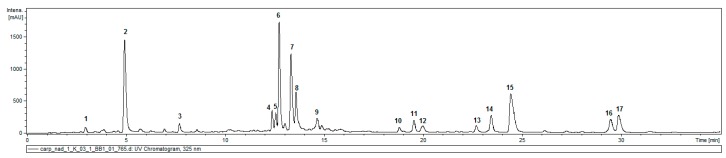
An HPLC-UV chromatogram of *Carpesium divaricatum* aerial part extract (10 mg/mL, 2 μL injected) acquired at 325 nm.

HPLC-DAD-MS^n^ analysis of hydroalcoholic extracts from aerial parts of the plant revealed the presence of 17 compounds with absorption maxima at 324–328 nm (typical of caffeic acid and its conjugates). No substantial peaks of compounds that demonstrated different UV/Vis spectral properties were observed (Figure 1). Peaks 1 and 2 (*m*/*z* = 353 [M − H]^−^) were easily identified as signals of 3-*O*-caffeoylquinic acid (3-CQA; IUPAC numbering system) and 5-*O*-caffeoylquinic acid (5-CQA), respectively, whilst compounds 4–6 and 8 (*m*/*z* = 515 [M − H]^−^), taking into consideration fragmentation patterns of their quasimolecular ions (Table 1), proved to be four different isomers of di-O-caffeoyl quinic acid (DCQA), namely: 3,4-; 1,5-; 3,5-; and 4,5-di-*O*-caffeoylquinic acids [8,9]. Peaks 3, 7, and 9, representing compounds that showed a cleavage of two or three caffeoyl [M – H − (2 − 3 × 162)]^−^ moieties resulting in the *m*/*z* 209 fragment, were assigned to hexaric acid derivatives: di-*O* caffeoylhexaric acid (I) (peak 3), tri-*O*-caffeoylhexaric acid (I) (peak 7), and tri-*O*-caffeoylheharic acid (II) (peak 9). The compounds have been previously described as constituents of other plants of the Asteraceae family [10,11,12,13], including the systematically related species *Inula helenium* [14]. Compounds corresponding to peaks 10 and 11 were tentatively identified as isobutyryl-dicaffeoylquinic acids based on the presence of quasimolecular ions at *m*/*z* 585 [M − H]^−^ and product ions at *m*/*z* 497 [585 − C_3_H_7_COOH]^−^, 423 [585 − caffeoyl]^−^, 335 [497 − caffeoyl]^−^ or [423 − C_3_H_7_COOH]^−^, and 179 [caffeic acid − H]^−^, which is in accordance with the data given by Heilmann et al. [15]. The compound represented by peak 12 (*m*/*z* at 677 [M − H]^−^) and producing fragmentation ions at *m*/*z* 515 and 353 was tentatively identified as 3,4,5-tri-*O*-caffeoylquinic acid [16]. Compounds corresponding to signals 13 and 14 (*m*/*z* at 599 [M − H]^−^), which demonstrated similar fragmentation patterns to those of 10 and 11, except for the fact that the product ion at *m*/*z* 497 was generated by the cleavage of 2-methylbutyryl or 3-methylbutyryl (isovaleryl) instead of the isobutyryl moiety [599 − C_4_H_9_COOH]^−^, could easiest be envisioned as 2-methylbutyryl/isovaleryl-di-*O*-caffeoylquinic acids. The compound represented by the signal 15 was probably a deoxy derivative of leontopodic acid judging by the *m*/*z* value of its quasimolecular ion (765 [M − H]^−^) and fragmentation ions at *m*/*z* 603 and 441. The fragmentation pattern of leontopodic acid (*m*/*z* = 781 [M − H]^−^) exhibited fragment ions at *m*/*z* = 619 [M − H − caffeoyl]^−^ and at *m*/*z* = 457 [M − H − 2 × caffeoyl]^−^ [17]. Finally, the compounds corresponding to peaks 16 and 17, with molecular ion masses and fragmentation ion masses being 14 mass units higher than those of 15, could be tentatively identified as 2-methylbutyryl/isovaleryl-tri-*O*-caffeoylhexaric acids. The dry plant material contained 1.23 ± 0.006% of 5-CQA and 1.46 ± 0.023% of 3,5-DCQA.

### 2.2. Identification of Trans-12-Oxo-phytodienoic Acid (trans-12-OPDA)

**Figure 2:**
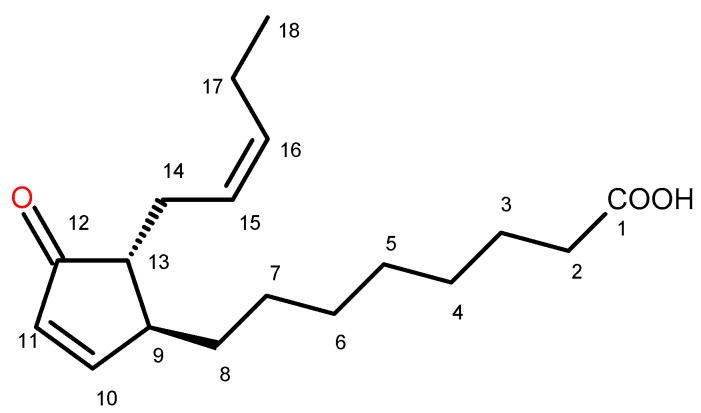
The chemical structure of (+)-trans-12-oxo-phytodienoic acid (trans-12-OPDA) isolated from aerial parts of Carpesium divaricatum Sieb. & Zucc.

Chromatographic separation of the chloroform extract from aerial parts of *C. divaricatum*, except for the mixtures of sesquiterpene lactones, which are known constituents of the plant [7], gave several fractions containing substantial amounts of the compound that showed a UV absorption maximum at 221 nm (HPLC-DAD). After multiple fractionation by means of chromatographic methods, the compound was isolated and identified, on the basis of its spectral data (see Section 4.5.), as *trans*-12-oxo-phytodienoic acid (12-OPDA, Figure 2).

### 2.3. Effect of trans-12-OPDA on Lipopolysaccharide (LPS)-Stimulated Release of Pro-Inflammatory Cytokines from Human Neutrophils

#### 2.3.1. Cytotoxicity

**Figure 3:**
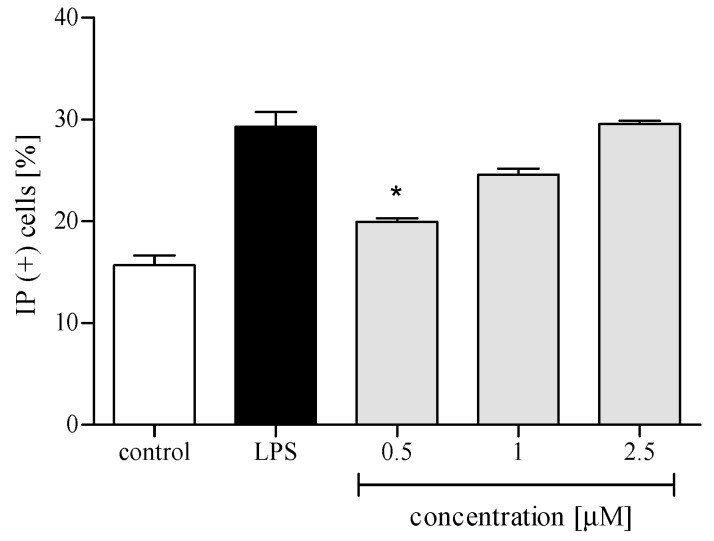
Cytotoxic effects of *trans*-12-OPDA, at concentrations of 0.5, 1.0, and 2.5 μM, on lipopolysaccharide (LPS)-stimulated human neutrophils. Results are shown as percent of cells with diminished membrane integrity (propidium-iodide-positive cells, PI (+)). Control, untreated cells; LPS, cells stimulated with LPS (stimulated control). Statistical significance * *p* < 0.05, with reference to a stimulated control.

Cytotoxicity of *trans*-12-OPDA to human polymorphonuclear leukocytes (PMNs) was assessed using propidium iodide (PI) staining and flow cytometry (FACS) analysis. The compound was essentially nontoxic in PMNs at 2.5 μM or lower concentrations (Figure 3). Solvent DMSO did not induce any cytotoxicity to the cells (data not shown). On the basis of these data, all further experiments were performed using *trans*-12-OPDA at concentrations up to 2.5 μM.

#### 2.3.2. Reactive Oxygen Species (ROS) Production

**Figure 4:**
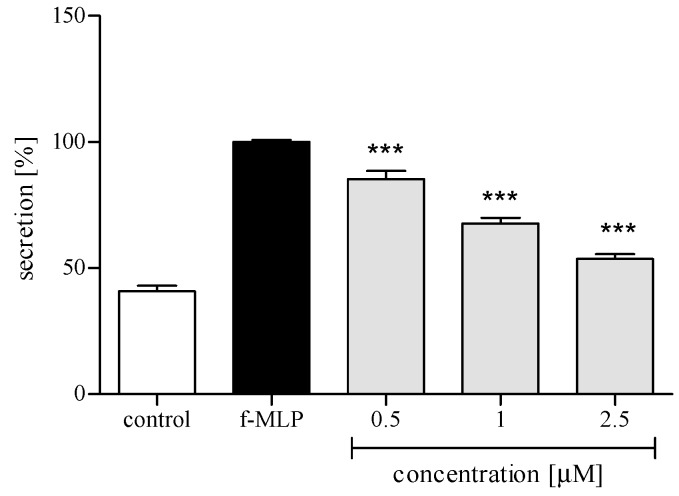
Inhibitory effects of *trans*-12-OPDA, at concentrations of 0.5, 1.0, and 2.5 μM, on reactive oxygen species (ROS) release from f-MLP-stimulated human neutrophils. Statistical significance *** *p* < 0.001, with reference to a stimulated control.

Activation of PMNs at a site of inflammation induces an oxidative burst in these cells. The phenomenon is characterized by intense ROS production and liberation of proteolytic enzymes from azurophilic granules. An effect of *trans*-12-OPDA on ROS generation in PMNs was examined in response to cytochalasin A/N-formyl-Met-Leu-Phe (f-MLP) stimulation. The examined oxylipin efficiently reduced ROS release at 1–2.5 μM (Figure 4).

#### 2.3.3. Release of Selected Proinflammatory Cytokines/Chemokines (IL-8, TNFα, IL-1β, CCL2)

**Figure 5:**
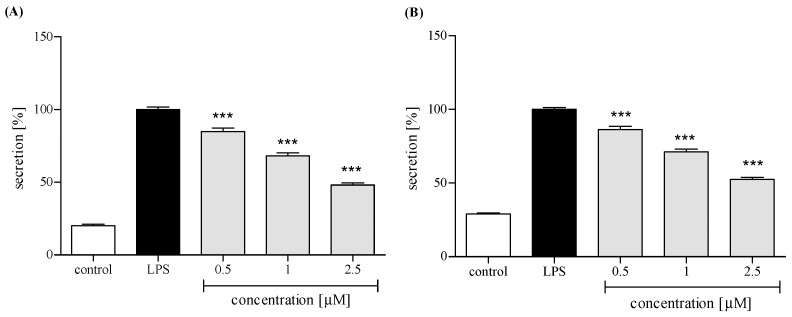
Inhibitory effects of *trans*-12-OPDA, at concentrations of 0.5, 1.0, and 2.5 μM, on IL-8 (**A**) and TNFα (**B**) secretion by LPS-stimulated human neutrophils. Statistical significance *** *p* < 0.001, with reference to a stimulated control.

**Figure 6:**
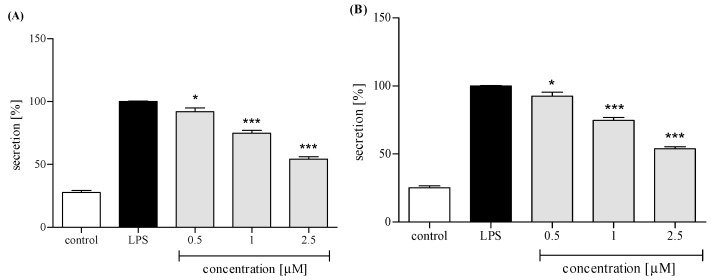
Inhibitory effects of *trans*-12-OPDA, at concentrations of 0.5, 1.0, and 2.5 μM, on IL-1β (**A**) and CCL2 (**B**) secretion by LPS-stimulated human neutrophils. Statistical significance: * *p* < 0.05, *** *p* < 0.001, with reference to a stimulated control.

Neutrophils, in response to stimulation with proinflammatory agonists, e.g., LPS or f-MLP, secrete an array of cytokines and chemokines, including TNFα, IL-1β, IL-8, and CCL2 [18,19]. Human neutrophils were pretreated with *trans*-12-OPDA before their priming with LPS. Using ELISA, we determined levels of IL-8, TNFα, IL-1β, and CCL2 in the culture medium 24 h after LPS stimulation. Preincubation of human neutrophils with *trans*-12-OPDA resulted in significant and dose-dependent inhibition of IL-8 production upon LPS stimulation (Figure 5A). The oxylipin was also active as an inhibitor of TNFα production. A statistically significant reduction in the release of this cytokine was achieved with all tested doses of the compound (see Figure 5B). Both IL-1β and CCL2 production by LPS-treated neutrophils were moderately and dose-dependently lowered by pretreatment with *trans*-12-OPDA at a dose of 0.5–2.5 μM (Figure 6A,B).

## 3. Discussion

Hydroxycinnamates, including chlorogenic acids, as common constituents of fruit, vegetables, and widely consumed beverages (coffee) are intrinsic components of the human diet with potential health-enhancing properties [20,21]. Some recent reports suggest their implication in prevention of so-called lifestyle diseases, such as diabetes [22,23], cardiovascular and neoplastic diseases [24,25], oxidative-stress-induced cognitive impairment [26,27,28,29], and obesity [30]. The Asteraceae plants are rich in hydroxycinnamates representing different structural types [21,31]. Plants of the Inuleae tribe are also good producers of acylquinic acids [31,32,33]. Despite their proven value as active plant metabolites, hydroxycinnamates have remained unexplored constituents of *Carpesium* sp. [3]. In the hydroalcoholic extract from aerial parts of *C. divaricatum*, we managed to detect 17 caffeic acid derivatives, using the HPLC-DAD-MS^n^ method. Seven of them, including two monocaffeoylquinic acids, four dicaffeoylquinic acids (DCQAs), and 3,4,5-tri-*O*-caffeoylquinic acid, are quite common within the Asteraceae. Their chromatographic behavior and mass fragmentation patterns have been thoroughly studied before. Thus, their identification was not challenging. Conjugates of caffeic acid with hexaric acid (or acids) have been less investigated. Referring to our previous study, on caffeic acid derivatives from tissue culture of *Inula helenium* [14], we were able to identify three further compounds as dicaffeoylhexaric acid ([M − H]^−^ 533) and two tricaffeoylhexaric acids ([M − H]^−^ 695). The compounds were formerly reported as constituents of the Asteraceae plants belonging to the tribes Eupatorieae [11], Gnaphalieae [10,12], and Millerieae of the Heliantheae alliance [13,34]. While investigating phenolic compounds from flowerheads of *Buphthalmum salicifolium* L. (Asteraceae, Inuleae), Heilmann and coworkers [15] isolated 1-isobutyryl-3,5-DCQA. Its molecular ion—586 [M]^+^ and fragmentation pattern in negative ion mode nicely corresponded to those of the compound eluted at 19.8 min. The compound of the same molecular mass, eluted at 19.0 min, is very likely another isobutyryl ester of DCQA. Taking into consideration that esterification of both terpenoid and phenolic metabolites with isobutyric acid and/or 2-methylbutyric/isovaleric acid is quite common within Inuleae [7,35,36], we postulated that the compounds eluted at 22.9 min and 23.8 min were 2-methylbutyryl/isovaleryl-DCQAs. Finally, on the basis of spectral data of leontopodic acid, given by Schwaiger et al. [17], we tentatively identified the three remaining compounds as isobutyryl-tricaffeoylhexaric acid (*m*/*z* at 765 [M − H]^−^) and 2-methylbutyryl/isovaleryl-tricaffeoylhexaric acids (*m*/*z* at 779 [M − H]^−^). The estimated contents of 5-CQA (1.23% dry weight) and 3,5-DCQA (1.46% dry weight), together with the relation of peak areas of the two compounds to the areas of remaining hydroxycinnamate signals visible on the chromatogram, allow us to conclude that aerial parts of *C. divaricatum* could be a rich dietary source of these compounds [37].

12-OPDA is an intermediate in jasmonic acid biosynthesis in plants. The compound itself demonstrates physiological activity as a hormone regulating seed dormancy, embryo development, redox homeostasis, and response to stress [38]. As an optically active molecule, 12-OPDA may exist in four possible absolute configurations (9*S*,13*S*; 9*S*,13*R*; 9*R*,13*R*; and 9*R*,13*S*). On the basis of ^1^H-NMR spectral data, we were able to exclude *cis* orientation of side chains in our compound (i.e., 9*S*,13*S* and 9*R*,13*R* configurations) [39]. *Cis* or *trans* orientation of the side chains in 12-OPDA molecule can be easily determined by comparison of the chemical shift values for protons at C-8, C-9, and C-13 of the compound with those reported by Baertschi et al. [39]. Two protons at C-8 of the *cis* isomer give rise to two multiplets at *δ_H_* 1.15 and 1.73 (1H each), whereas both protons at C-8 of the *trans*-12-OPDA give one signal at *δ_H_* 1.49 (2H). Signals of protons at C-9 and C-13 of *trans*-12-OPDA are shifted upfield of those in the *cis*-OPDA by c. 0.4 ppm. Discrimination between the 9*S*,13*R trans* isomer and the 9*R*,13*S trans* isomer was possible by optical activity measurement. The dextrorotary activity of 12-OPDA isolated by us (see Section 4.5.) proved that the compound was (9*S*,13*R*)-12-OPDA, i.e., (+)-*trans*-12-OPDA [40]. Except for the activity as a plant hormone, 12-OPDA exerted cytoprotective effects in human neuroblastoma SH-SY5Y cells subjected to oxidative stress [41], mitigated LPS-induced inflammation in microglia [42], inhibited proliferation of human breast cancer cells [43], and demonstrated antileishmanial activity [44]. Thus, we decided to check whether or not the compound may contribute to the overall activity of *C. divaricatum*.

PMNs are an essential part of human immune system. They are, among other things, engaged in the inflammation process, which could be a consequence of bacterial infection, environmental stress, or some kinds of neoplastic disease. The neutrophils, except for performing phagocytosis, generating toxic molecules, releasing enzymes, and forming extracellular traps, participate in fine regulation of the immune response and inflammatory process via a capability to respond to and to produce a variety of cytokins [19]. Cytokins produced by human neutrophils include the proinflammatory cytokine IL-1β, TNFα, and the chemokines CCL2 and IL-8 (CXCL8). In order to establish whether or not *trans*-12-OPDA may influence neutrophil function, we decided to monitor secretion of the mentioned cytokines by LPS-stimulated human neutrophils in the absence or presence of the compound. LPS stimulation of neutrophils is mediated via toll-like receptors (TLRs), and this process includes activation of both the NF-κB and mitogen-activated protein (MAP) kinase pathways. At a site of inflammation, leucocytes are activated and, subsequently, an intense production of ROS by the cells takes place. We observed that incubation of human neutrophils with 12-OPDA significantly and in a concentration-dependent manner diminished f-MLP–induced ROS production by the cells (Figure 4). The examined oxylipin significantly reduced secretion of proinflammatory cytokines (IL-1β, TNFα) and chemokines CCL2 and IL-8 by LPS-stimulated human neutrophils (Figure 5 and Figure 6). The inhibitory effect exerted by the lowest concentration tested (0.5 μM) was slightly weaker towards IL-1β and CCL2 secretion (*p* < 0.05, Figure 6). The results of our tests suggested that 12-OPDA may be implicated in the anti-inflammatory activity of *C. divaricatum* preparations. Effects observed in vitro nicely corresponded to the traditional use of the plant.

In conclusion, we would like to emphasize that the aerial parts of *C. divaricatum* are a rich and still not completely explored source of bioactive molecules. The variety of their chemical structures and bioavailability after ingestion are far from being completely investigated. Though sesquiterpene lactones isolated from the plant have proven activity as cytotoxic and anti-inflammatory agents, they are not the sole compounds responsible for the pharmacological activity of the plant. The set of specialized metabolites produced by *C. divaricatum* suggests considerable potential for the future application of the plant either as a functional food or as an herbal medicinal product.

## 4. Materials and Methods

### 4.1. General Methods

Mass spectra were recorded using a MALDI-TOF/TOF ultrafleXtreme mass spectrometer (Bruker Daltonics, Billerica, MA, USA). ^1^H-NMR spectra were recorded in CDCl_3_ on a Bruker AVANCE III HD 400 (resonance frequency 400.17 MHz) spectrometer. Optical rotation was determined in CDCl_3_ on a PolAAr31 polarimeter (Optical Activity Ltd., Ramsey, England, UK). RP-HPLC separations were performed using an Agilent 1200 Series HPLC system (Agilent Technologies, Santa Clara, CA, USA) equipped with a diode array detector. Analytical chromatographic separations were carried out on a Kinetex XB-C18 column (4.6 × 250 mm, 5 μm total particle size; Phenomenex, Torrance, CA, USA). Semipreparative RP-HPLC was conducted on a Synergi 4μ Fusion-RP, 80A, 250 × 10 mm column (Phenomenex, Torrance, CA, USA), with an isocratic elution, using MeOH-H_2_O mixtures of different polarities. Conventional column chromatography was carried out on Silica gel 60 (0.063–0.2 mm, Merck, Darmstadt, Germany). TLC separations were performed using precoated plates (Silica gel 60 without fluorescence indicator, Art. No 5553, Merck, Germany).

### 4.2. Materials

Chlorogenic acid (5-*O*-CQA, purity > 97% by HPLC) and a standard sample of cynarin (1,3-DCQA, purity > 99% by HPLC) were purchased from Roth (Karlsruhe, Germany). Organic solvents of analytical grade were purchased either from POCh S.A. (Gliwice, Poland) or from Merck (Darmstadt, Germany). Water was purified by a Mili-Q system (Milipore Corp., Bedford, MA, USA). MeOH and MeCN of HPLC grade as well as formic acid and glacial acetic acid were purchased from Merck (Darmstadt, Germany).

Phosphate-buffered saline (PBS) was bought from Biomed (Lublin, Poland). Hanks’ balanced salt solution (HBSS), cell culture medium (RPMI 1640 medium), f-MLP (formyl-Met-Leu-Phenylalanine), LPS (from *Escherichia coli* 0111:B4), propidium iodide (PI), luminol, 4-(2-hydroxyethyl)-1-piperazineethanesulfonic acid (HEPES) solution, and l-glutamine were purchased from Sigma-Aldrich Co. (St. Louis, MO, USA). Fetal bovine serum (FBS) was delivered by Gibco (Grand Island, NY, USA).

### 4.3. Plant Material

*C. divaricatum* plants were grown in the Garden of Medicinal Plants, Institute of Pharmacology, Polish Academy of Sciences, Kraków from seeds that were provided by the Research Center for Medicinal Plant Resources, National Institute of Biomedical Innovation (Tsukuba, Japan). Aerial parts of the plants were collected in the beginning of flowering period (August/September) and dried under shade at room temperature. A voucher specimen (3/15) was deposited in the collection kept at the Garden of Medicinal Plants, Institute of Pharmacology, Kraków, Poland.

### 4.4. Analysis of Caffeic Acid Derivatives

#### 4.4.1. Extract Preparation and Characterization by the HPLC-DAD-MS^n^ Method

Dried and pulverized aerial parts of *C. divaricatum* (0.1 g) were extracted twice with 10 mL of 70% MeOH at room temperature, for 3 h, on a rotary shaker (100 r.p.m.). The extracts were combined and evaporated to dryness under reduced pressure to give a residue. The obtained residue (10 mg) was dissolved in a mixture of MeOH and 0.1% HCOOH (8:2, *v*/*v*) and then filtered through a 0.45 μm Chromafil membrane (Machery-Nagel, Duren, Germany). HPLC-DAD-MS^n^ analysis was performed on a UHPLC-3000 RS system (Dionex, Germany) with a photodiode array (DAD) detection and an AmaZon SL ion trap mass spectrometer with an ESI interface (Bruker Daltonics GmbH, Germany). Separation was performed on a Kinetex XBC18 column (150 × 2.1 mm, 1.7 μm) Phenomenex (Torrance, CA, USA). The column temperature was 25 ºC. The mobile phase (A) was water/formic acid (100:0.1, *v*/*v*) and the mobile phase (B) was acetonitrile/formic acid (100:0.1, *v*/*v*). A gradient system was used: 0–10 min 10–25% B; 10–40 min 25–35% B. The flow rate was 0.3 mL/min. The column was equilibrated for 7 min between injections. UV spectra were recorded over a range of 200–450 nm, and chromatograms were acquired at 325 nm. The LC eluate was introduced directly into the ESI interface without splitting. The nebuliser pressure was 40 psi; dry gas flow 9 L/min; dry temperature 300 °C; and capillary voltage 4.5 kV. Analysis was carried out using a scan from *m*/*z* 90 to 2200. Compounds were analyzed in a negative ion mode. The MS^2^ fragmentation was obtained for the most abundant ion at the time.

#### 4.4.2. Quantification of Chlorogenic Acid (5-CQA) and 3,5-di-*O*-caffeoylquinic Acid (3,5-DCQA)

The contents of 5-CQA and 3,5-DCQA, in the aerial parts of *C. divaricatum*, were estimated by means of the HPLC-DAD method, as was described earlier [14].

### 4.5. Isolation of Trans-12-Oxo-Phytodienoic Acid (12-OPDA)

Dry, pulverized shoots of *C. divaricatum* (267 g) were extracted four times with CHCl_3_ (1.5 L). The organic extracts were combined and concentrated in vacuo to provide 24 g of an oily residue. The residue was fractionated by conventional column chromatography (CC) on silica gel using an *n*-hexane–EtOAc gradient solvent system (up to 100% EtOAc). Collected fractions (50 mL each) were combined as shown by TLC. Fractions 175–181, eluted with *n*-hexane–EtOAc 7:3 (*v*/*v*), were subjected to semiprep. RP-HPLC (eluent: MeOH–H_2_O mixture, 4:1, *v*/*v*, isocratic mode, flow rate: 2 mL/min) to yield *trans*-12-OPDA (13.5 mg, purity > 92% by HPLC).

#### (+) trans-12-Oxo-phytodienoic acid: (9S,13R) OPDA

Amorphous solid: [α]_D_^25.6^: +69.3 (c = 0.5, CHCl_3_); UV (MeCN-H_2_O) λ_max_ 221 nm; ^1^H-NMR (400.17 MHz, CDCl_3_): δ 7.62 (1H, dd, *J* = 5.7, 2.4 Hz, H-10), 6.15 (1H, dd, *J* = 5.7, 1.8 Hz, H-11), 5.47 (1H, m, H-16), 5.27 (1H, m, H-15), 2.59 (1H, m, H-9), 2.47 (1H, m, H-14a), 2.37 (2H, t, *J* = 7.4, H-2a, H-2b), 2.31 (1H, m, H-14b), 2.07 (2H, m, H-17a, H-17b), 2.04 (1H, m, H-13), 1.65 (2H, m, H-3a, H-3b), 1.50 (2H, m, H-8a, H-8b), 1.27–1.40 (8H, m, H-4a, H-4b, H-5a, H-5b, H-6a, H-6b, H-7a, H-7b), 0.98 (3H, t, *J* = 7.4). MALDI-MS/MS (pos. mode) m/z: 293.2 [C_18_H_28_O_3_ + H]^+^; calc. 293.2.

### 4.6. Isolation of Human Neutrophils

Peripheral venous blood was taken from healthy human donors (18–35 years old) in the Warsaw Blood Donation Centre. Donors did not smoke or take any medications. They were clinically recognized to be healthy and routine laboratory tests showed all values to be within the normal ranges. The study conformed to the principles of the Declaration of Helsinki. Neutrophils were isolated by dextran sedimentation and centrifugation in a Ficoll Hypaque gradient and then resuspended in (Ca^2+^)-free HBSS buffer or RPMI 1640 medium.

### 4.7. Assessment of the Effects of Trans-12-OPDA on Lipopolysaccharide (LPS)-Stimulated Release of Pro-Inflammatory Cytokines from Human Neutrophils

#### 4.7.1. Cytotoxicity Measurement

Cytotoxicity was assessed by a standard flow cytometric probe using propidium iodide (PI) staining. After 24 h of incubation in the absence or presence *trans*-12-OPDA (at concentrations of 0.5, 1.0, 2.5, or 5.0 μM), the neutrophils (3.5 × 10^5^) were harvested and centrifuged (1500 r.p.m.; 10 min; 4 °C), washed once with cold PBS, and resuspended in 400 μL of PBS. A 5 μL aliquot of PI solution (50 μg/mL) was added to the cell suspension. After 15 min of incubation with PI at room temperature, cells were analyzed by a BD FACSCalibur flow cytometer (BD Biosciences, San Jose, CA, USA), and 10,000 events were recorded per sample. The number of cells that displayed high permeability to PI, expressed as a percentage of PI (+) cells, was determined.

#### 4.7.2. ROS Production by Neutrophils

ROS production was measured using a luminol-dependent chemiluminescence test. A 70 μL aliquot of neutrophil suspension (3.5 × 10^5^) in (Ca^2+^)-free HBSS buffer, 50 μL of the tested compound solution, and 50 μL of luminol (100 μM) were added to a well in a 96-well plate. ROS production was initiated by the addition of f-MLP (30 μL of 0.1 μg/mL solution) to obtain a total volume of 200 μL per well. Chemiluminescence changes were measured for 40 min, at 2 min intervals, in a microplate reader (37 °C). The background chemiluminescence produced by non-stimulated cells was also determined. The percentage of inhibition was calculated by comparison to the stimulated control without the tested compound, at the maximum luminescence.

#### 4.7.3. IL-8, IL-1β, CCL-2, and TNFα Production by Neutrophils

Neutrophils (2 × 10^6^) were cultured in 24-well plates in RPMI 1640 medium with 10% FBS, 10 mM HEPES, and 2 mM L-glutamine, in the presence or absence of LPS (100 ng/mL) and in the absence or presence of *trans*-12-OPDA (final concentration in a range of 0.5–5 μM), at 37 °C with 5% CO_2_. After 24 h, the neutrophils were harvested and centrifuged (2000 r.p.m.; 10 min; 4 °C). The amount of released cytokines was measured by enzyme-linked immunosorbent assay (ELISA) following the manufacturer’s instructions (BD Biosciences, USA). The effects on IL-8, IL-1β, CCL2, and TNFα production were calculated by comparing the percentages of the released agents to the stimulated control, which lacked the test compound.

### 4.8. Statistical Analysis

The results were expressed as the mean ± standard error of the mean (SEM) of three independent experiments performed at least in duplicate. All analyses were performed using Statistica 13 software. The statistical significance of the differences between means was established by ANOVA with Dunnett’s post hoc test *p* values.

## Figures and Tables

**Table 1:** Retention times, UV maxima, and MS^n^ data, in the negative ion mode, for the phenolic compounds present in extracts from *Carpesium divaricatum* aerial parts.

Compound		Retention Time (min)	UV (nm)	[M − H]^−^	Product Ions Main Peaks ^1^
1	3-*O*-caffeoylquinic acid	3.2	325	353	**191**, 179
2	5-*O*-caffeoylquinic acid	5.1	325	353	**191**
3	Dicaffeoylhexaric acid (I)	7.9	324	533	**371**, 209
4	3,4-di-*O*-caffeoylquinic acid	12.6	325	515	**353**, 335, 299, 255, 203, 191, 179, 173
5	1,5-di-*O*-caffeolyquinic acid	12.8	328	515	**353**, 335, 191
6	3,5-di-*O*-caffeoylquinic acid	13.0	327	515	**353**, 191
7	tricaffeoylhexaric acid (I)	13.5	327	695	**533**, **371**, 209
8	4,5-di-*O*-caffeoylquinic acid	13.8	327	515	**353**, 317, 299, 255, 203, 173
9	tricaffeoylhexaric acid (II)	14.8	328	695	**533**, **371**, 209
10	isobutyryl-dicaffeoylquinic acid	19.0	326	585	**497**, 335, 317, 299, 255, 179
11	isobutyryl-dicaffeoylquinic acid	19.8	328	585	497, **423**, 335, 179
12	tri-*O*-caffeoylquinic acid	20.1	327	677	**515**, 353
13	2-methylbutyryl/isovaleryl-dicaffeoylquinic acid	22.9	326	599	**497**, 335, 299, 179
14	2-methylbutyryl/isovaleryl-dicaffeoylquinic acid	23.8	328	599	497, **437**, 335, 179
15	isobutyryl-tricaffeoylhexaric acid	24.7	328	765	**603**, 441
16	2-methylbutyryl/isovaleryl-tricaffeoylhexaric acid	29.7	327	779	**617**, 455
17	2-methylbutyryl/isovaleryl-tricaffeoylhexaric acid	30.2	327	779	**617**, 455

**^1^** MS^2^ ions in bold are the most abundant ion peak.
